# Toward Intelligent Emergency Triage: A Feasibility Study of Real-Time Facial Expression-Based Chest Pain Intensity Assessment

**DOI:** 10.3390/diagnostics16091346

**Published:** 2026-04-29

**Authors:** Yu-Tse Tsan, Rita Wiryasaputra, Yi-Jun Hsieh, Qi-Xiang Zhang, Hsing-Hung Liu, Chao-Tung Yang

**Affiliations:** 1 School of Medicine, Chung Shan Medical University, Taichung 402306, Taiwan; janyuhjer@gmail.com; 2 Department of Emergency Medicine, Institute of Occupational Medicine, Taichung Veterans General Hospital, Taichung 407219, Taiwan; ph0422ph0422@gmail.com; 3 Department of Occupational Safety and Health Office, Taichung Veterans General Hospital, Taichung 407219, Taiwan; 4 Department of Post-Baccalaureate Medicine, College of Medicine, National Chung Hsing University, Taichung 407224, Taiwan; 5 Informatics Department, Krida Wacana Christian University, Jakarta 11470, Indonesia; rita.wiryasaputra@ukrida.ac.id; 6 Department of Computer Science, Tunghai University, Taichung 407224, Taiwan; 7 Institute of Computer Science and Engineering, National Yang Ming Chiao Tung University, Hsinchu 300093, Taiwan; 8 Department of Emergency Medicine, Kuang Tien General Hospital, Taichung 433004, Taiwan; 9 Research Center for Smart Sustainable Circular Economy, Tunghai University, Taichung 407224, Taiwan; 10 Department of Medical Research, Kuang Tien General Hospital, Taichung 433004, Taiwan

**Keywords:** deep learning, face detection, expression recognition, image recognition, YOLO

## Abstract

**Objectives**: Ensuring an effective triage to treat patients with chest pain in emergency settings is critical, but it can often be challenging, particularly when patients wear face masks or are unable to clearly communicate their pain. To address this limitation, this study presents a real-time facial expression–based system for chest pain intensity assessment as an initial step toward realizing intelligent emergency triage. The proposed system integrates deep learning with real-time video analysis to provide objective and rapid pain level recognition. **Methods**: A YOLOv12-based facial expression recognition model was trained using annotated facial images of patients experiencing chest pain, and the model categorizes pain into three intensity levels: no pain, slight pain, and moderate to severe pain. Multiple YOLOv12 variants were systematically evaluated to identify an optimal configuration for potential clinical use. The developed system supports two operational modes: real-time recognition, which analyzes continuous video streams and delivers immediate visual feedback through an interactive interface, and a manual upload mode for offline video analysis, review of results, and playback. Additional usability features, including error prompts and data reset functions, were implemented to enhance system stability and user experience. **Results**: Among the evaluated models, the YOLOv12-L model achieved the best performance with an accuracy of 98.81%, sensitivity of 98.76%, specificity of 98.79%, precision of 98.04%, and an F1-score of 98.41%, demonstrating stable and accurate recognition. The proposed system is designed to support the triage process of assessing patients with chest pain, particularly in cases where patients wear masks or cannot clearly express their pain. By providing real-time and objective pain intensity assessment, the system shows potential to assist healthcare professionals in identifying patients who may require priority attention and to serve as a supportive tool for emergency triage workflows. **Conclusions**: Future work will incorporate edge computing with a lightweight model to enable real-time pain assessment in ambulances, facilitating faster intervention and treatment.

## 1. Introduction

Although Taiwan has entered an aging society [1], accompanied by persistently low birth rates that continue to strain healthcare systems, the country has also made advancements in medical technology and improvements in public health. These demographic shifts have led to a substantial increase in emergency department (ED) utilization, where older adults—often presenting with complex, acute, and multi-morbid conditions—now constitute a growing proportion of visits. As ED overcrowding intensifies, triage nurses are required to rapidly assess the severity of each patient under significant time pressure. This increases the risk of subjective judgment, inconsistent evaluations, and delayed recognition of life-threatening symptoms.

Chest pain is one of the most critical ED presentations due to its strong association with acute coronary syndrome, myocardial infarction, and other emergent cardiovascular conditions. Accurate and timely assessment of chest pain intensity is therefore essential for effective risk categorization. However, conventional pain evaluation methods rely primarily on patient self-reporting or verbal communication, which may be unreliable among older adults, cognitively impaired patients, individuals with communication barriers, or those experiencing severe distress. These limitations highlight the need for objective, real-time, and non-invasive tools to support clinical triage.

Facial expressions provide a rich and quantifiable source of information for pain assessment. Facial Expression Recognition (FER) has emerged as a promising approach for automated pain assessment, with growing applications in emotion analysis, behavior prediction, and healthcare [2,3,4]. In clinical pain research, the Prkachin and Solomon Pain Intensity Coding System (PSPI) is widely used to quantify pain-related facial movements based on specific Action Units (AU) [5]. However, PSPI relies on manual frame-by-frame annotation, limiting its applicability in real-time emergency settings.

Pain-related facial expressions have been systematically characterized using the Facial Action Coding System (FACS), where specific Action Units (AU) such as brow lowering, orbital tightening, and upper lip raising are consistently associated with nociceptive responses. However, chest pain–related expressions may involve additional complexity beyond general pain responses. In emergency contexts, chest pain is often accompanied by physiological distress and anxiety, leading to combined expressions of pain and affective tension. Compared to other emotional states such as fear or sadness, these expressions tend to exhibit stronger and more sustained facial muscle activation, reflecting both physical discomfort and cardiovascular urgency. These characteristics suggest that chest pain–related facial expressions may be distinguishable from other affective states.

Recent advances in deep learning have enabled efficient and scalable facial expression analysis [6,7,8]. Convolutional Neural Networks (CNNs) are fundamental for visual classification and object detection tasks [9], showing effectiveness in pain and emotion recognition [10,11]. Among existing approaches, the You Only Look Once (YOLO) family is particularly suitable for real-time applications due to its high inference speed [12,13]. Recent developments, such as YOLOv12, further improve feature representation and multi-scale learning, enabling better capture of subtle facial cues related to pain intensity [14,15]. In this study, YOLOv12 is adapted as a unified framework that performs both facial region detection and pain-level classification in a single stage, where each detected face is directly assigned a corresponding pain intensity label.

Despite these advances, real-time deep learning approaches that are specifically designed for chest pain–related facial expression recognition for emergency triage settings remain limited. Existing approaches often rely on controlled datasets, involve high computational cost, or lack robustness in dynamic clinical environments.

To address this gap, this study proposes a YOLOv12-based real-time facial expression analysis framework for chest pain intensity assessment. The proposed system is designed as a decision-making support tool that provides an objective and instantaneous estimation of pain severity, particularly in situations where verbal communication is limited. This work focuses on evaluating the technical and clinical feasibility of integrating deep learning–based facial analysis into emergency triage workflows, laying an initial foundation for future intelligent triage systems. The main contributions of this study are as follows:

1. Construct a real-time YOLOv12-based facial expression recognition model capable of identifying facial features associated with chest pain intensity.
2. Conduct a comprehensive performance comparison across multiple YOLOv12 variants to determine an optimal configuration for a potential clinical setting.
3. Develop a clinically-aligned chest pain classification workflow designed to support ED triage through rapid and objective pain assessment.
4. Explore the technical feasibility of integrating the proposed system into real-world healthcare settings, providing an initial foundation for future intelligent triage emergency solutions.

This study extends our previous works on pain recognition by incorporating more advanced YOLO architectures, such as YOLOv11 and YOLOv12, and focusing on real-time, clinically relevant chest pain assessment within emergency triage workflows. The article is organized as follows: Section 2 provides a review of related work, materials, and research methodology are presented in Section 3, while Section 4 covers the experimental results. Section 5 presents a discussion. Finally, the conclusion and future work are presented in Section 6.

## 2. Related Work

Early studies on automated pain recognition primarily relied on CNN architectures trained on the UNBC dataset, focusing on binary or coarse multi-level pain classification [16,17]. Subsequent studies introduced attention mechanisms and multi-scale feature learning to improve sensitivity to localized facial cues, achieving four-level pain classification using enhanced CNN variants such as multi-scale regional attention networks (MSRAN) and global–local attention networks [18,19]. Several approaches have also explored hybrid and multimodal frameworks that combine facial expressions with physiological or behavioral signals, particularly on the BioVid Heat Pain dataset [20,21,22]. While these methods demonstrate improved representational capacity, they often depend on additional sensors and controlled acquisition environments, limiting their practicality for real-time emergency department deployment. More recent studies have adopted object detection–based architectures, such as YOLOv8, to enable faster inference and simplified pipelines [23]. However, these approaches typically focus on binary pain classification and general pain scenarios rather than clinically aligned, multi-level chest pain assessment. Overall, existing studies are constrained by limited pain level granularity, reliance on controlled datasets, or computational complexity that hinders real-time application. These limitations highlight a critical gap for a lightweight, vision-only, and real-time pain intensity recognition framework tailored specifically to emergency triage settings, spurring the need to use YOLOv12 for the present investigation. Our previous studies explored binary and multi-level pain classification using earlier YOLO variants [24,25]. However, these works primarily focused on model benchmarking and did not address real-time deployment or clinical integration in emergency settings. The present study builds upon this foundation by introducing more advanced architectures and a clinically oriented framework. Prior studies on automated facial pain intensity recognition have explored a wide range of modeling strategies, datasets, and classification granularities, as summarized in Table 1.

## 3. Materials and Methods

**Figure 1:**
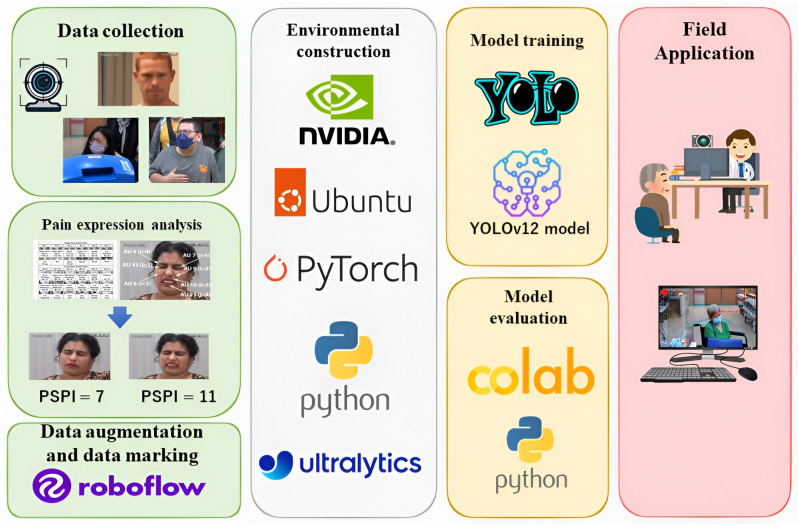
Proposed system framework.

**Figure 2:**
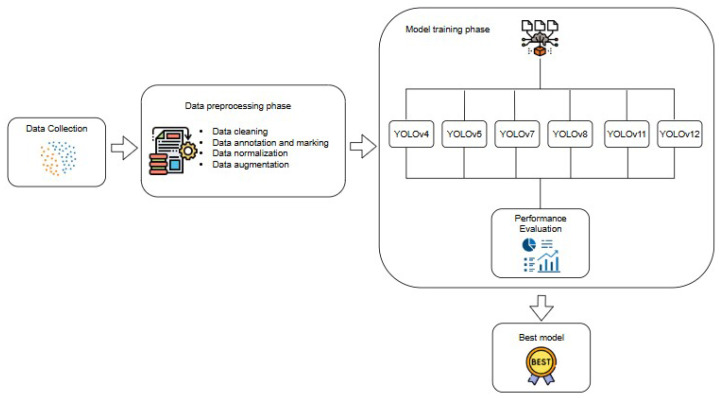
Workflow diagram.

This section provides a comprehensive overview of our approach, including the data collection, preprocessing, model implementation, and performance evaluation. The proposed system framework, as illustrated in Figure 1, comprises four main stages: data processing, environment setup, model training, and field application, while Figure 2 depicts the corresponding workflow.

### 3.1. Data Collection

This study employed two datasets to ensure the robustness and clinical relevance of the proposed system. The first dataset, the UNBC-McMaster Shoulder Pain Archive [30], contains facial expression image sequences from 25 participants exhibiting varying levels of shoulder pain. Each frame is annotated with PSPI scores. Although the UNBC-McMaster dataset focuses on shoulder pain, it captures core facial muscle activation patterns related to pain expression, which have been widely adopted in pain-related facial analysis and are applicable for model pretraining and methodological validation [17,19,31,32,33,34,35,36,37].

**Figure 3:**
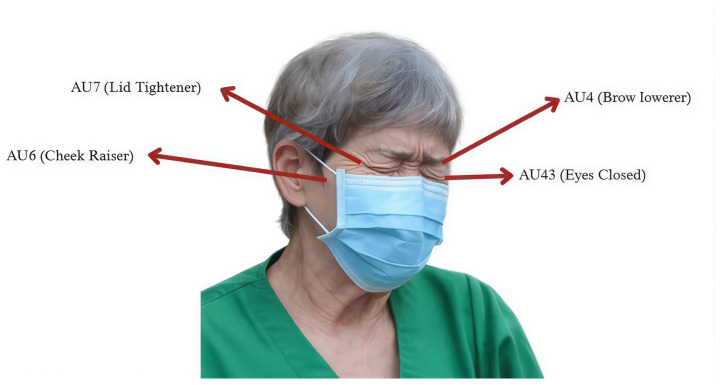
Facial Action Coding System (FACS) with Action Units (AUs).

The PSPI formulation is grounded in the FACS, which encodes facial muscle movements into discrete AU. Figure 3 illustrates the FACS framework and its corresponding AU, which were used for facial expression analysis.

The PSPI scale (defined in Equation (1)) represents a discrete ordinal measure of pain intensity, ranging from no pain (0) to maximum pain (16) [5,30]. The specific AU used to calculate PSPI, along with their relevance to pain-related facial movements, are summarized in Table 2. For clinical interpretation, PSPI scores were grouped into three severity levels. Level 1 (no-pain; PSPI = 0) corresponds to neutral facial expressions. Level 2 (slight pain; PSPI = 1–6) is characterized by mild pain-related facial cues. Level 3 (severe pain; PSPI = 7–16) reflects pronounced pain expressions associated with strong facial muscle activation.

(1) $$\begin{array}{c} PSPI_{\mathrm{score}}=\mathrm{AU}4+\max\left(\mathrm{AU}6,\mathrm{AU}7\right)+\max\left(\mathrm{AU}9,\mathrm{AU}10\right)+\mathrm{AU}43 \end{array}$$

In post-pandemic clinical settings, however, patients commonly wear face masks that occlude the nose and mouth area. Such occlusion significantly limits the reliable detection of AU9 and AU10. Therefore, these AU were excluded from the masked-face analysis, and the PSPI scoring formulation was accordingly adapted, as shown in Equation (2).

(2) $$\begin{array}{c} PSPI_{\mathrm{score}}=\mathrm{AU}4+\max\left(\mathrm{AU}6,\mathrm{AU}7\right)+\mathrm{AU}43 \end{array}$$

The second dataset comprises self-collected video recordings of patients presenting with chest pain, obtained from the emergency department of Taichung Veterans General Hospital (TVGH). To extract facial expressions, one frame was sampled every ten frames, converting the videos into static images while preserving temporal variability. All extracted frames were manually reviewed and annotated by medical professionals into three pain levels—no pain, slight pain, and severe pain—ensuring the reliability and accuracy of the training data. Data collection adhered to ethical standards, received approval from the Institutional Review Board, and all facial data were handled in compliance with privacy policies and encrypted as required.

### 3.2. Data Preprocessing

Both datasets underwent standardized preprocessing, including data cleaning, annotation, normalization, and augmentation. Offline augmentation techniques such as rotation, horizontal flipping, and brightness perturbation were applied prior to training to expand the dataset and improve model generalization. Global and local contrast normalization were employed to mitigate the effects of occlusion, lighting variability, and skin tone differences. The Roboflow platform was used for data annotation and marking, including face-level bounding box labeling and pain-level classification, as well as image resizing to standardize input resolution. This preprocessing strategy further reduced the impact of mask-wearing by emphasizing upper-face regions, particularly the eyebrows and eyes, which are critical for pain-related facial expression analysis.

In total, 48,398 images were compiled and initially organized into twelve folders corresponding to PSPI levels 0–11 based on a masked-PSPI definition adapted for occluded facial regions. During data inspection, approximately 70% of samples initially auto-labeled as PSPI = 0 were identified as incorrect due to the misdetection of facial AU under poor lighting conditions or extreme head poses. These samples were subjected to secondary expert review, and only images containing valid facial cues were retained for subsequent model development.

Clinical consultation at TVGH indicated that the original 12-level PSPI scale does not align well with the five-level emergency triage system commonly used in practice. In addition, facial expressions alone provide limited discriminative power at fine-grained pain levels. To improve clinical relevance and usability, the PSPI-based labels were consolidated into three categories: no pain (PSPI = 0), mild pain (PSPI = 1–4), and moderate to severe pain (PSPI = 5–11). This unified three-level labeling scheme was applied consistently across both datasets, enabling integrative model training while maintaining compatibility with real-world clinical workflows.

**Figure 4:**
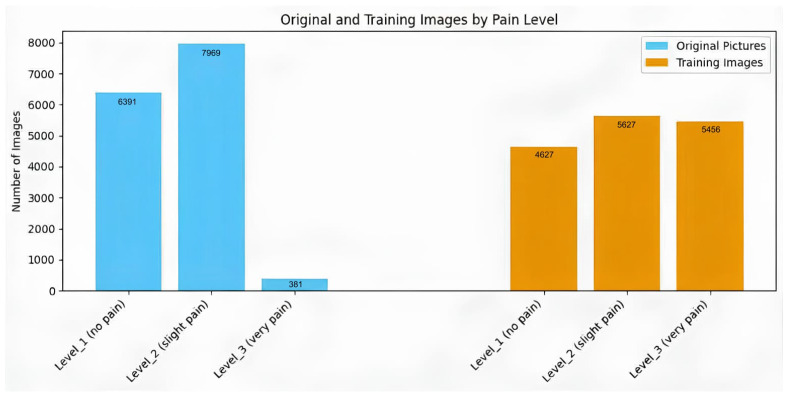
Distribution of original and training images across pain levels.

The final dataset includes both masked and non-masked facial images with sufficient representation across the three severity levels. Sample distribution is illustrated in Figure 4.

### 3.3. Environment Setup and Runtime Configuration

The experiments were conducted on a workstation equipped with an NVIDIA RTX 4090 GPU running the Linux Ubuntu 22.04 operating system. The development environment utilized Python 3.10.12 with a PyTorch 2.3.0-based YOLO training pipeline. GPU acceleration was enabled through compute unified device architecture (CUDA) 12.2 and CUDA deep neural network (cuDNN) library. Under this hardware configuration, the system was capable of processing YOLO-based facial expression recognition at an input resolution of 640 × 640 pixels, achieving an average inference speed of approximately 20 frames per second (FPS). This processing rate satisfies the real-time requirements of emergency triage scenarios. To improve temporal stability and reduce frame-level prediction noise, recognition results were aggregated over a 1-s interval (20 consecutive frames). If more than 80% of the frames within a given interval produced the same classification result, that pain level was considered dominant and displayed as an indicator in the top-left corner of the video stream. In cases where the combined proportion of Level 2 (slight pain) and Level 3 (severe pain) exceeded 80%, but neither level individually met the threshold, the system selected the level with the higher frame count. If none of these conditions were satisfied, the system deferred the decision and continued accumulating additional frames.

### 3.4. Model Training

The dataset was partitioned into training, validation, and test sets using a 70%:20%:10% ratio, respectively. The training set was used for model optimization, the validation set for performance monitoring and hyperparameter tuning, and the test set for final evaluation. Model training was conducted for 300 epochs using input images resized to 640 × 640 pixels, with a batch size of 8. These hyperparameters were selected to achieve a balance between detection accuracy and computational efficiency, given the memory constraints and real-time deployment considerations. This configuration reflects a practical compromise between model performance and training efficiency, supporting the feasibility of deploying the proposed system in real-time emergency triage environments.

### 3.5. Model Evaluation

The model’s predictive performance was evaluated using a confusion matrix, which summarizes the relationship between the predicted outputs and the true labels. The matrix categorizes outcomes into True Positive (TP), False Positive (FP), False Negative (FN), and True Negative (TN), providing a clear overview of classification behavior. To ensure that each chest pain level is fairly evaluated, macro-average metrics were computed for each class individually, and then the unweighted mean across all classes was taken. The key detection metrics—including accuracy (Equation (3)), sensitivity (Equation (4)), specificity (Equation (5)), precision (Equation (6)), and F1-score (Equation (7))—were computed to comprehensively assess the model’s accuracy and robustness.

(3) $$\begin{array}{c} {\mathrm{Accuracy}}_{\mathrm{macro}\text{-}\mathrm{average}}=\frac{1}{N}\sum_{i=1}^{N}\frac{TP_{i}+TN_{i}}{TP_{i}+TN_{i}+FP_{i}+FN_{i}} \end{array}$$

(4) $$\begin{array}{c} {\mathrm{Sensitivity}}_{\mathrm{macro}\text{-}\mathrm{average}}=\frac{1}{N}\sum_{i=1}^{N}\frac{TP_{i}}{TP_{i}+FN_{i}} \end{array}$$

(5) $$\begin{array}{c} {\mathrm{Specificity}}_{\mathrm{macro}\text{-}\mathrm{average}}=\frac{1}{N}\sum_{i=1}^{N}\frac{TN_{i}}{TN_{i}+FP_{i}} \end{array}$$

(6) $$\begin{array}{c} {\mathrm{Precision}}_{\mathrm{macro}\text{-}\mathrm{average}}=\frac{1}{N}\sum_{i=1}^{N}\frac{TP_{i}}{TP_{i}+FP_{i}} \end{array}$$

(7) $$\begin{array}{c} F1\text{-}{\mathrm{score}}_{\mathrm{macro}\text{-}\mathrm{average}}=\frac{1}{N}\sum_{i=1}^{N}2\times \frac{{\mathrm{Precision}}_{i}\times {\mathrm{Sensitivity}}_{i}}{{\mathrm{Precision}}_{i}+{\mathrm{Sensitivity}}_{i}} \end{array}$$

### 3.6. Field Application

**Figure 5:**
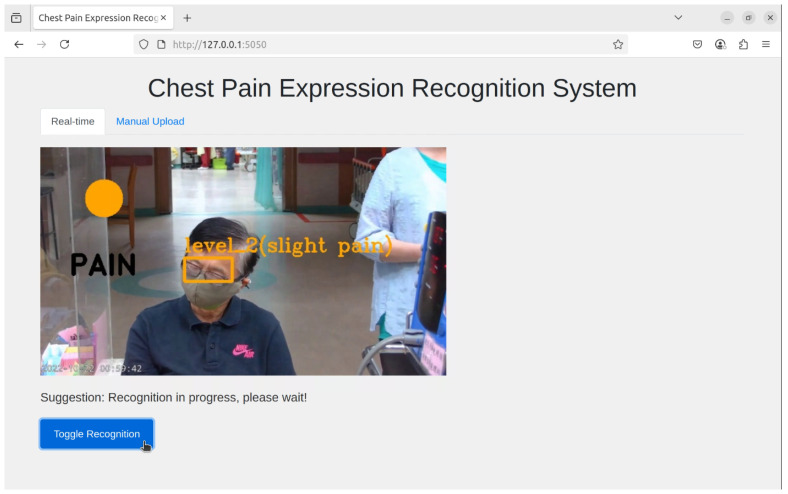
User interface for real-time recognition.

**Figure 6:**
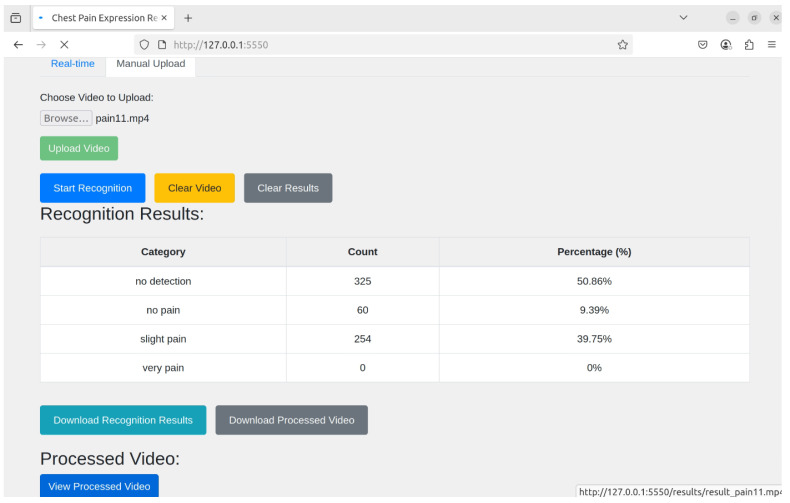
Data analysis.

The proposed system supports two operational modes—real-time recognition and manual upload (non-real-time) recognition—providing flexibility for different application scenarios. In the real-time mode, input is acquired directly from a camera, where streaming frames are continuously resized to meet the model’s input specifications and processed by the best-performing YOLO model. This enables immediate visualization of detection results, making it suitable for monitoring and time-sensitive applications, as shown in Figure 5. The overlapping annotations in the detection results represent concurrent predictions of multiple facial action units and do not indicate visualization errors. In contrast, the non-real-time mode allows users to upload images or video files, which are subsequently processed through a frame extraction stage to generate uniformly sized frames. These frames are then passed through the proposed YOLO-based inference pipeline, and the detection outputs are aggregated and compiled for further analysis. As illustrated in Figure 6, the results are summarized into three columns—category, count, and percentage—allowing users to efficiently evaluate the distribution and proportion of each pain category across the entire video. The aggregated results can also be downloaded, supporting retrospective analysis and batch processing. Although some elements appear visually overlapping in Figure 6, they are used solely for layout compactness and do not obscure any critical information or affect the interpretability of the proposed system.

## 4. Results

This study evaluates the performance of multiple YOLO-based object detection models for recognizing three levels of pain expression using facial imagery: no pain, slight pain, and severe pain. The YOLO versions adopted include YOLOv4, YOLOv5, YOLOv7, YOLOv8, YOLOv11, and YOLOv12, and they were initially compared to assess their capability in low-level pain discrimination.

As shown in Table 3, recognition performance for the no pain category varied notably across YOLO model versions. YOLOv4 achieved a confidence score of 0.96, while YOLOv5 misclassified the sample as slight pain. YOLOv7 and YOLOv8 failed to detect the target, whereas YOLOv11 successfully recognized the class with a confidence of 0.88. Among the six models, YOLOv12 produced the highest confidence score at 0.98, indicating superior recognition stability for low-intensity pain expressions.

**Figure 7:**
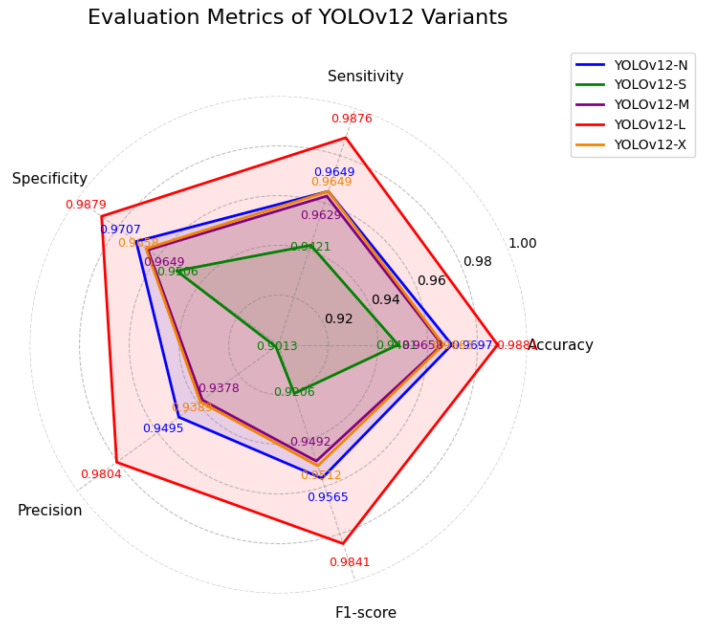
Macro-average evaluation metrics (Accuracy, Sensitivity, Specificity, Precision, and F1-score) for different YOLOv12 model variants.

Given the superior baseline performance of YOLOv12, further experiments focused on five YOLOv12 series (N, S, M, L, and X). The macro-average performance across these model scales is summarized in Table 4 and is visualized in Figure 7. These results indicate that while all YOLOv12 variants perform competitively, substantial differences exist in their ability to discriminate subtle pain expressions.

**Figure 8:**
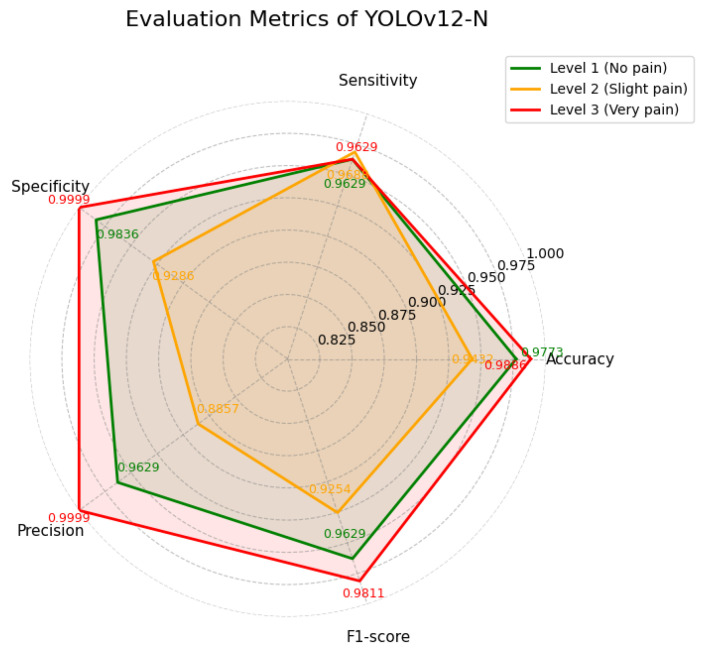
Evaluation metrics of YOLOv12-N.

**Figure 9:**
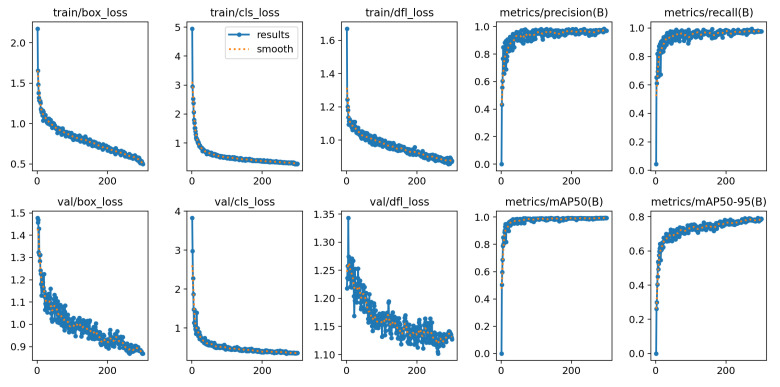
Analysis of YOLOv12-N training results.

YOLOv12-N achieved a high mean Average Precision (mAP) of 98.6%, indicating strong overall detection performance. As shown in Table 5 and is visualized in Figure 8, the model performs particulary well in distinguishing visually distinct categories, namely no pain and severe pain, with high precision and F1-scores. In contrast, lower precision is observed for the slight pain class compared to the other categories. As shown in Figure 9, YOLOv12-N exhibits stable training behavior, with decreasing training and validation losses across epochs. Precision, recall, and mAP metrics increase rapidly and stabilize at high values, indicating strong and consistent detection performance.

**Figure 10:**
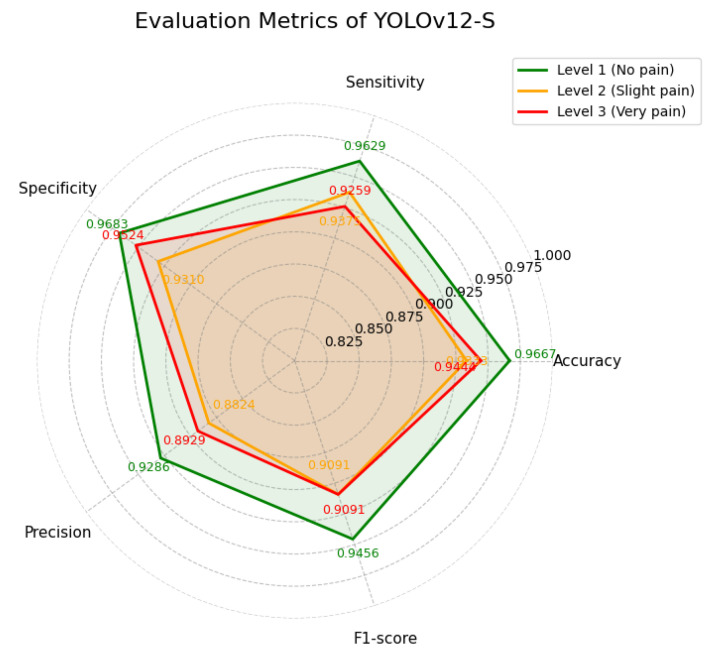
Evaluation metrics of YOLOv12-S.

**Figure 11:**
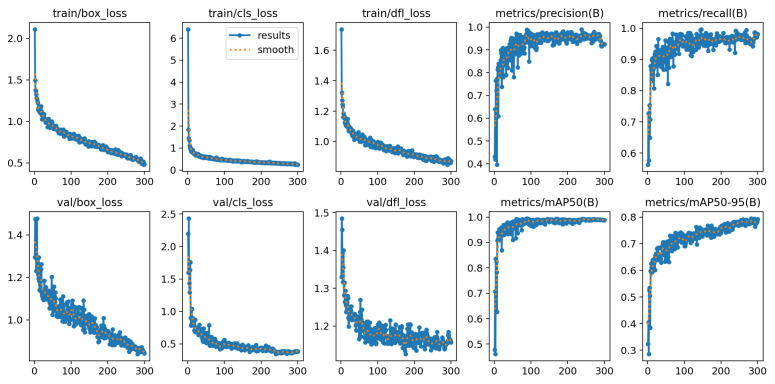
Analysis of YOLOv12-S training results.

YOLOv12-S achieved a slightly higher mAP of 98.9%, indicating strong overall detection performance. As reported in Table 6 and illustrated in Figure 10, the model maintains generally high classification performance across classes. However, its precision and F1-score for the slight pain category are the lowest among all variants, indicating reduced performance in Level 2 recognition. Figure 11 further shows stable training dynamics, with decreasing losses and consistently high evaluation metrics throughout training.

**Figure 12:**
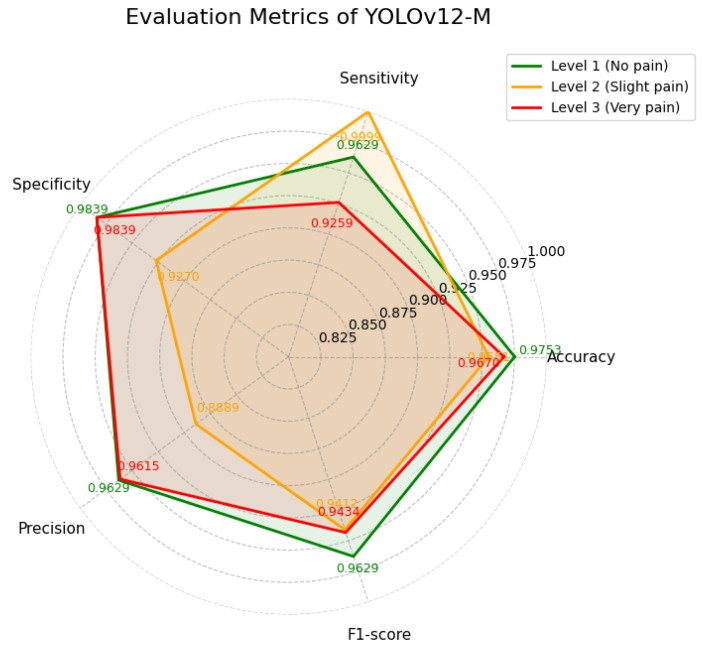
Evaluation metrics of YOLOv12-M.

**Figure 13:**
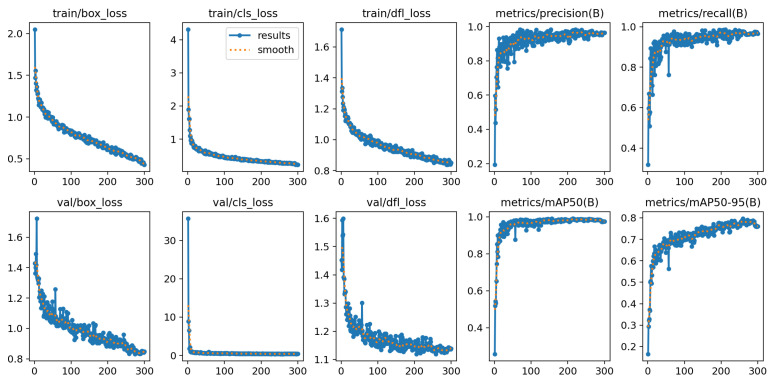
Analysis of YOLOv12-M training results.

YOLOv12-M achieved a mAP of 98.7%, demonstrating balanced performance across classes. As summarized in Table 7 and illustrated in Figure 12, the model shows particularly high sensitivity for Level 2 (slight pain), reaching 0.9999. In contrast, the sensitivity for Level 3 (very pain) is comparatively lower at 0.9259. The training loss curves of the YOLOv12-M model are shown in Figure 13, indicating stable convergence during training.

**Figure 14:**
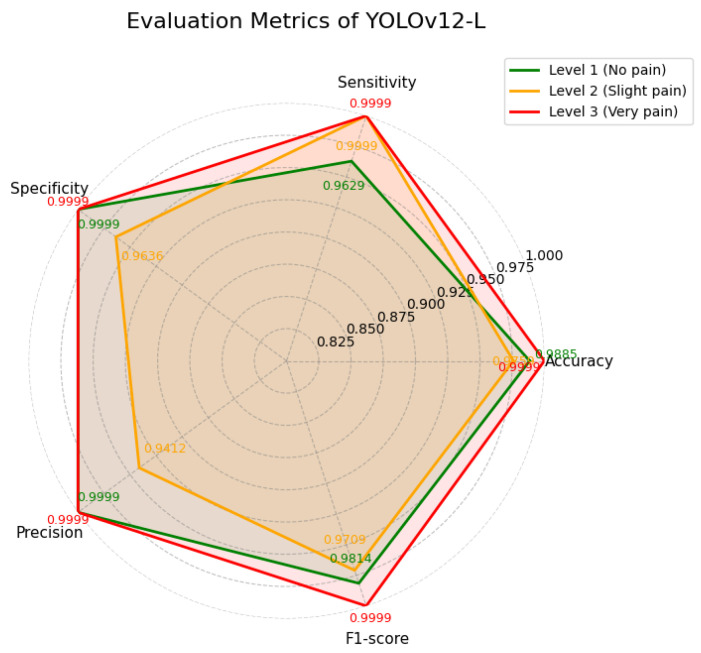
Evaluation metrics of YOLOv12-L.

**Figure 15:**
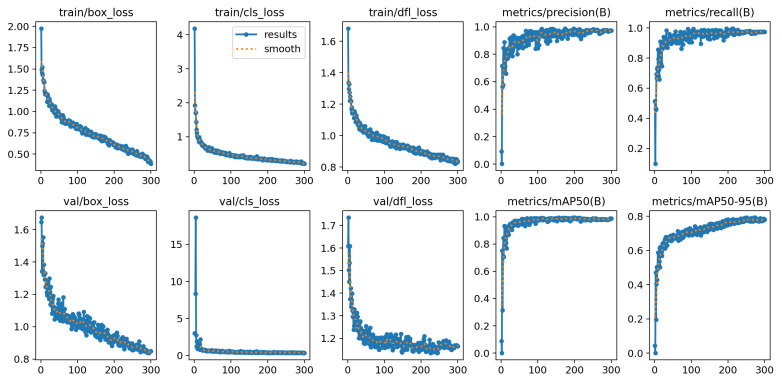
Analysis of YOLOv12-L training results.

YOLOv12-L achieved the highest overall mAP of 99.0% and demonstrated consistently strong performance across all three pain levels. As summarized in Table 8 and illustrated in Figure 14, the model maintained exceptionally high scores, with Level 3 (very pain) reaching near-perfect values (up to 0.9999) across all evaluation metrics. Levels 1 and 2 also exhibited high sensitivity and precision. Furthermore, the training loss curves shown in Figure 15 indicate stable convergence during training.

**Figure 16:**
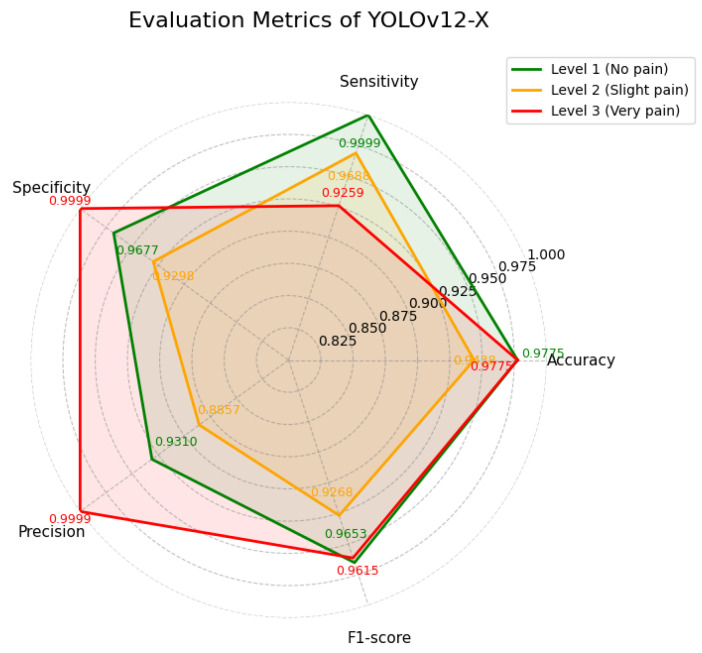
Evaluation metrics of YOLOv12-X.

**Figure 17:**
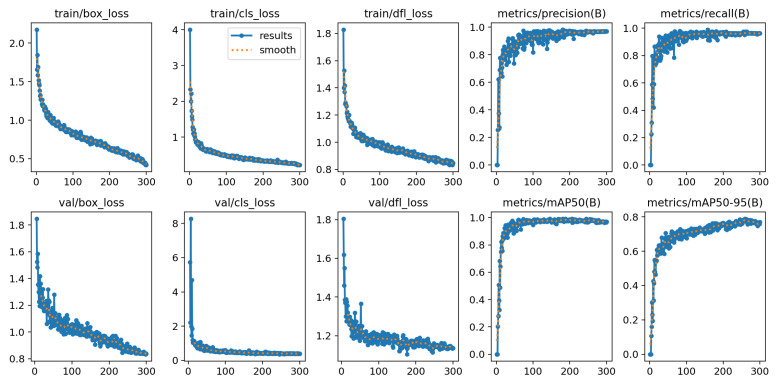
Analysis of YOLOv12-X training results.

YOLOv12-X achieved the same mAP as YOLOv12-N (98.6%), but exhibited different sensitivity–precision trade-offs across categories. As shown in Table 9 and Figure 16, the model attained very high sensitivity for Level 1 (0.9999), accompanied by lower precision (0.9310). For Level 3, precision reached 0.9999, while sensitivity was comparatively lower (0.9259). Additionally, the training curves is shown in Figure 17.

**Figure 18:**
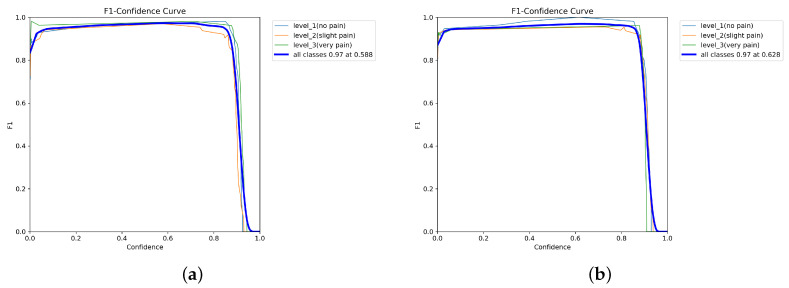
F1-score. (**a**) F1-confidence curve of YOLOv12-N. (**b**) YOLOv12-X.

**Figure 19:**
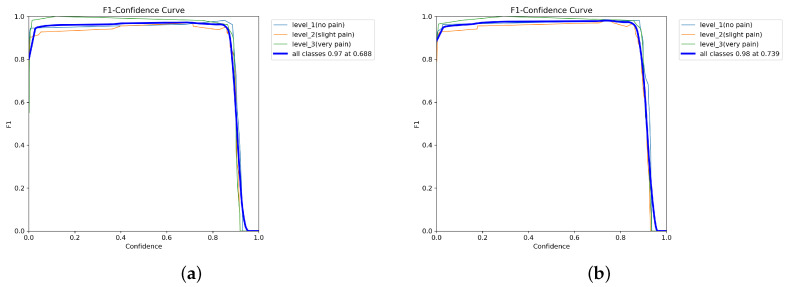
F1-score. (**a**) F1-confidence curve of YOLOv12-M. (**b**) YOLOv12-L.

As shown in Figure 18 and Figure 19, all YOLOv12 variants demonstrate stable F1–confidence behavior, with high F1-scores maintained across moderate confidence thresholds before declining at extreme values. YOLOv12-L achieves the highest peak F1-score, followed by YOLOv12-M, YOLOv12-X, and YOLOv12-N. Across all models, performance is consistently higher for no pain and very pain classes than for slight pain.

## 5. Discussion

**Figure 20:**
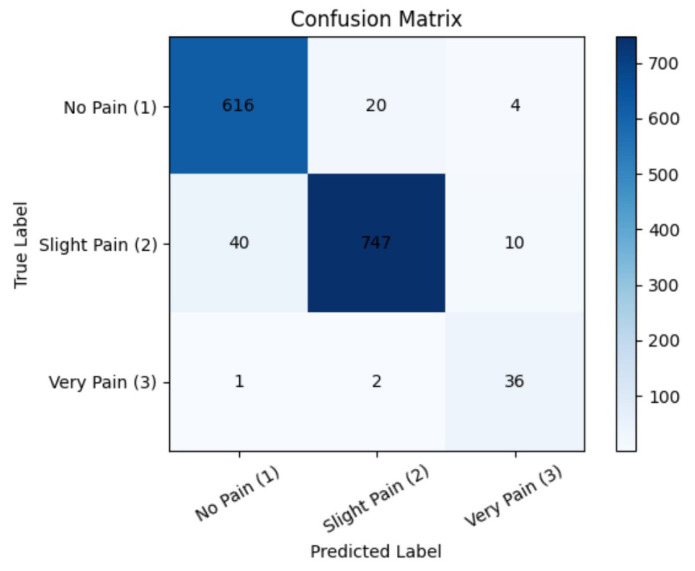
Confusion Matrix.

This study presents a comprehensive comparison of YOLOv12 variants for multi-level pain expression recognition, revealing that high overall accuracy does not necessarily translate into consistent performance across clinically relevant pain categories. Although all variants achieved mAP values exceeding 98%, their classification robustness varied substantially across pain levels, reflecting differences in architectural depth, feature extraction capacity, and sensitivity to subtle facial cues. A lightweight YOLOv12-N architecture demonstrated reliable detection of visually distinct expressions, particularly no pain and severe pain. However, their reduced representational capacity indicated limited performance for slight pain, which relies on subtle muscular changes and low-amplitude facial movements. From a clinical perspective, such limitations may result in underestimation of early-stage discomfort, potentially delaying timely intervention. YOLOv12-S showed marginal improvement in overall accuracy but remained inconsistent across classes. Its tendency to misclassify neutral expressions as pain suggests vulnerability to minor facial variations and environmental noise, which may negatively affect triage efficiency by increasing false-positive alerts. YOLOv12-M achieved a more balanced performance, particularly excelling in sensitivity for slight pain detection. This indicates enhanced responsiveness to subtle facial cues, although at the cost of increased false positives. While such behavior may be acceptable for screening-oriented systems, reduced sensitivity to severe pain warrants caution in safety-critical applications. As illustrated in Figure 20, the model demonstrates robust and reliable performance, particularly in distinguishing between no pain and slight pain conditions. Among all the models evaluated, YOLOv12-L achieves the most consistent performance across pain levels. Its deeper architecture and enhanced feature aggregation enable effective capture of both global facial structure and localized micro-expressions, which is particularly important in masked or partially occluded scenarios commonly encountered in emergency settings. Although YOLOv12-X achieved high precision, its comparatively lower sensitivity suggests a risk of missed detections, which is less desirable in an emergency triage where false negatives carry greater clinical risk than false positives. Overall, the balanced per-class performance proved more clinically significant than marginal gains in overall mAP.

From a feasibility perspective, the results suggest that facial expression–based pain intensity recognition can serve as a complementary decision-making tool in an emergency triage rather than a standalone diagnostic system. Models with balanced sensitivity across pain levels, such as YOLOv12-L, show potential for real-time inference scenarios, where early detection of potential severe pain is prioritized over marginal gains in overall accuracy. In practical triage workflows, such systems could assist clinicians by providing an initial, objective pain estimation when verbal communication is limited or delayed. Importantly, the observed trade-off between sensitivity and precision highlights the need to align model selection with clinical risk tolerance, where minimizing false negatives is critical. These findings provide preliminary evidence supporting the potential integration of deep learning–based facial analysis into intelligent emergency triage systems as an assistive, real-time screening layer, while further validation in real-world clinical settings remains necessary.

Despite the strong performance achieved, this study has several limitations. First, the dataset lacks sufficient diversity, which may limit the generalizability of the model across different age groups, ethnic backgrounds, and real-world clinical conditions. In addition, the dataset was partitioned using a random sample-based strategy rather than a subject-level split. Consequently, samples from the same individual may appear across the training, validation, and test sets, potentially introducing data leakage and leading to optimistic performance estimates. This may affect the model’s ability to generalize to unseen subjects. Second, the proposed framework primarily relies on facial expression–based pain detection as an early diagnostic support tool. From a clinical perspective, pain intensity alone does not reliably correlate with accurate diagnosis or prognosis. While effective for capturing observable pain-related features, such signals cannot fully represent the complexity of underlying physiological conditions. This limitation is particularly evident in scenarios such as cardiovascular abnormalities, where critical physiological responses—including sympathetic nervous system activation—may occur independently of visible pain expressions. The absence of multimodal physiological signals, therefore, constrains the system’s ability to reflect a comprehensive clinical state. Incorporating such signals would enable more accurate detection of latent physiological stress and improve clinical decision support. Relying solely on pain levels is insufficient for accurate clinical assessment.

## 6. Conclusions and Future Work

Multiple YOLOv12-based models were developed and systematically evaluated for chest pain expression classification to identify the most effective architecture for recognizing facial cues corresponding to varying pain levels. YOLOv12-L outperformed other variants, achieving 98.76% sensitivity and a 98.41% F1-score, suggesting its effectiveness in capturing subtle facial expression differences and assessing pain-related features under challenging conditions. The proposed model was integrated into a clinically aligned chest pain classification workflow, designed as a potential non-invasive decision-making support tool for rapid and objective patient triage in the ED. The system may contribute to improving triage efficiency, particularly for patients wearing masks or those who are unable to verbally communicate their symptoms. Overall, this work represents a step toward bridging the gap between theoretical model development and its potential clinical applicability.

Future work will focus on optimizing computational efficiency for edge deployment, expanding the dataset to include more diverse demographic and clinical profiles, and integrating multimodal information—including physiological signals, speech cues, patient history, and indicators of autonomic activation. The measure is to further enhance model accuracy, robustness, and generalizability. Overall, this study contributes to the advancement of intelligent emergency triage by demonstrating how real-time deep learning–based facial expression analysis can support clinical decision-making and improve patient care in time-critical settings.

## Figures and Tables

**Table 1:** Summary of related studies on facial pain intensity recognition.

Authors	Levels	Dataset	Model
[16]	n.a	UNBC	CNN
[24]	2	UNBC + self-collected	YOLO variant
[25]	3	UNBC + self-collected	YOLO variant
[17]	4	UNBC	CNN
[18]	4	UNBC	MSRAN
[19]	4	UNBC	Global–Local Attention CNN
[26]	2	UNBC	HOA-based Deep CNN
[27]	4	UNBC, DISFA	DarkNet19
[28]	4	UNBC	PFFM
[20]	5	BioVid Heat Pain	Multimodal
[29]	n.a	BioVid Heat Pain, AI4Pain	Multimodal
[23]	2	Delaware, KDEF, RaFD, Roboflow	YOLOv8
[21]	2	BioVid Heat Pain + self-collected	Multimodal
[22]	2	BioVid Heat Pain	Multimodal

**Table 2:** Action Units and corresponding facial movements.

Action Units (AU)	Facial Movement Description
AU4	Brow Lowerer
AU6	Cheek Raiser
AU7	Lid Tightener
AU9	Nose Wrinkler
AU10	Upper Lip Raiser
AU43	Eyes Closed

**Table 3:** Pain expression recognition from various YOLO version.

Version	Level 1	Level 2	Level 3
YOLOv4	0.96	0.99	0.97
YOLOv5	misclassified (slight pain)	0.82	misclassified (no pain)
YOLOv7	failed	0.55	failed
YOLOv8	failed	failed	failed
YOLOv11	0.88	failed	0.91
YOLOv12	0.98	0.99	0.99

**Table 4:** Macro-average evaluation metrics across different model scales of YOLOv12.

	Accuracy	Sensitivity	Specificity	Precision	F1-Score
YOLOv12-N	0.9697	0.9649	0.9707	0.9495	0.9565
YOLOv12-S	0.9481	0.9421	0.9506	0.9013	0.9206
YOLOv12-M	0.9658	0.9629	0.9649	0.9378	0.9492
YOLOv12-L	0.9881	0.9876	0.9879	0.9804	0.9841
YOLOv12-X	0.9663	0.9649	0.9658	0.9389	0.9512

**Table 5:** YOLOv12-N evaluation metrics.

	Accuracy	Sensitivity	Specificity	Precision	F1-Score
Level 1 (no pain)	0.9773	0.9629	0.9836	0.9629	0.9629
Level 2 (slight pain)	0.9432	0.9688	0.9286	0.8857	0.9254
Level 3 (very pain)	0.9886	0.9629	0.9999	0.9999	0.9811

**Table 6:** YOLOv12-S evaluation metrics.

	Accuracy	Sensitivity	Specificity	Precision	F1-Score
Level 1 (no pain)	0.9667	0.9629	0.9683	0.9286	0.9456
Level 2 (slight pain)	0.9333	0.9375	0.9310	0.8824	0.9091
Level 3 (very pain)	0.9444	0.9259	0.9524	0.8929	0.9091

**Table 7:** YOLOv12-M evaluation metrics.

	Accuracy	Sensitivity	Specificity	Precision	F1-Score
Level 1 (no pain)	0.9753	0.9629	0.9839	0.9629	0.9629
Level 2 (slight pain)	0.9551	0.9999	0.9270	0.8889	0.9412
Level 3 (very pain)	0.9670	0.9259	0.9839	0.9615	0.9434

**Table 8:** YOLOv12-L evaluation metrics.

	Accuracy	Sensitivity	Specificity	Precision	F1-Score
Level 1 (no pain)	0.9885	0.9629	0.9999	0.9999	0.9814
Level 2 (slight pain)	0.9759	0.9999	0.9636	0.9412	0.9709
Level 3 (very pain)	0.9999	0.9999	0.9999	0.9999	0.9999

**Table 9:** YOLOv12-X evaluation metrics.

	Accuracy	Sensitivity	Specificity	Precision	F1-Score
Level 1 (no pain)	0.9775	0.9999	0.9677	0.9310	0.9653
Level 2 (slight pain)	0.9438	0.9688	0.9298	0.8857	0.9268
Level 3 (very pain)	0.9775	0.9259	0.9999	0.9999	0.9615

## Data Availability

Dataset is available on request from the authors.
